# Supplementary figures and images for: Personalized logical models to investigate cancer response to BRAF treatments in melanomas and colorectal cancers
Source: PLoS Comput Biol. 2021 Jan 28;17(1):e1007900. doi: 10.1371/journal.pcbi.1007900 (PMC7872233; doi:10.1371/journal.pcbi.1007900)

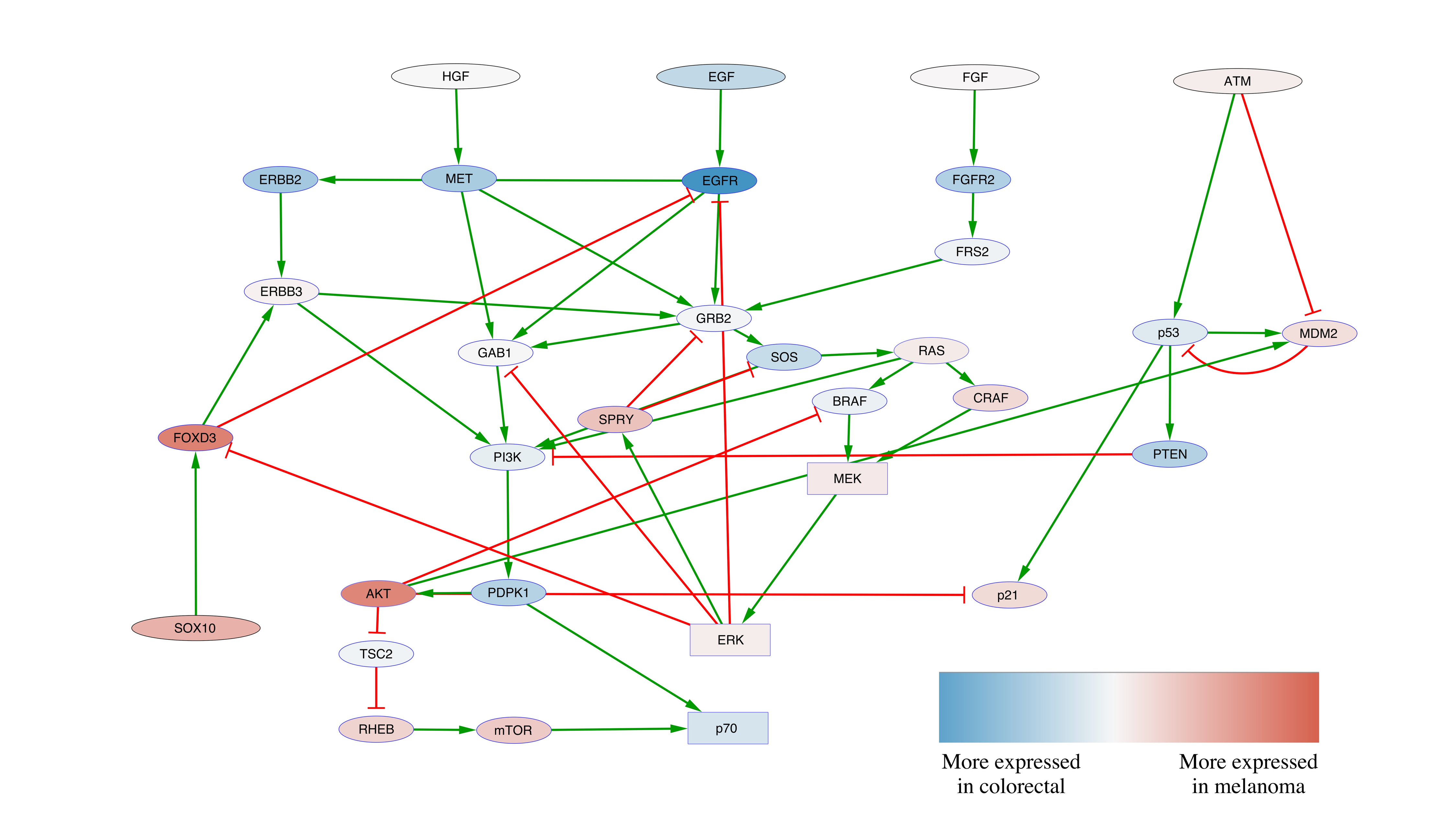

Supplement: S1 Fig — The expression data from both melanoma and colorectal cell lines used in this study are mapped onto the network. The scores correspond to the difference in the mean expression of the normalized data (using PROFILE method [35]). Red nodes show higher gene expression in melanomas and blue nodes higher expression in colorectal cancer cell lines. If most active nodes are equivalent to phosphorylated data, the mapping of RNAseq data informs on the gene status and the possibilities to activate the nodes. Thus, conclusions should be made with this fact in mind. At the gene level, then, genes such as SOX10, FOXD3, AKT, p21 and SPRY tend to have a higher expression in melanomas confirming their role in response to the treatment, whereas genes such as EGFR, ERBB2, MET, PTEN and FGFR2 are more relavant to colorectal cancers. This figure reinforces the idea that the mechanisms related to the response to anti-BRAF treatment may have different outcomes in both cancers bascule of a different gene context. (TIF) [file pcbi.1007900.s001.tif]

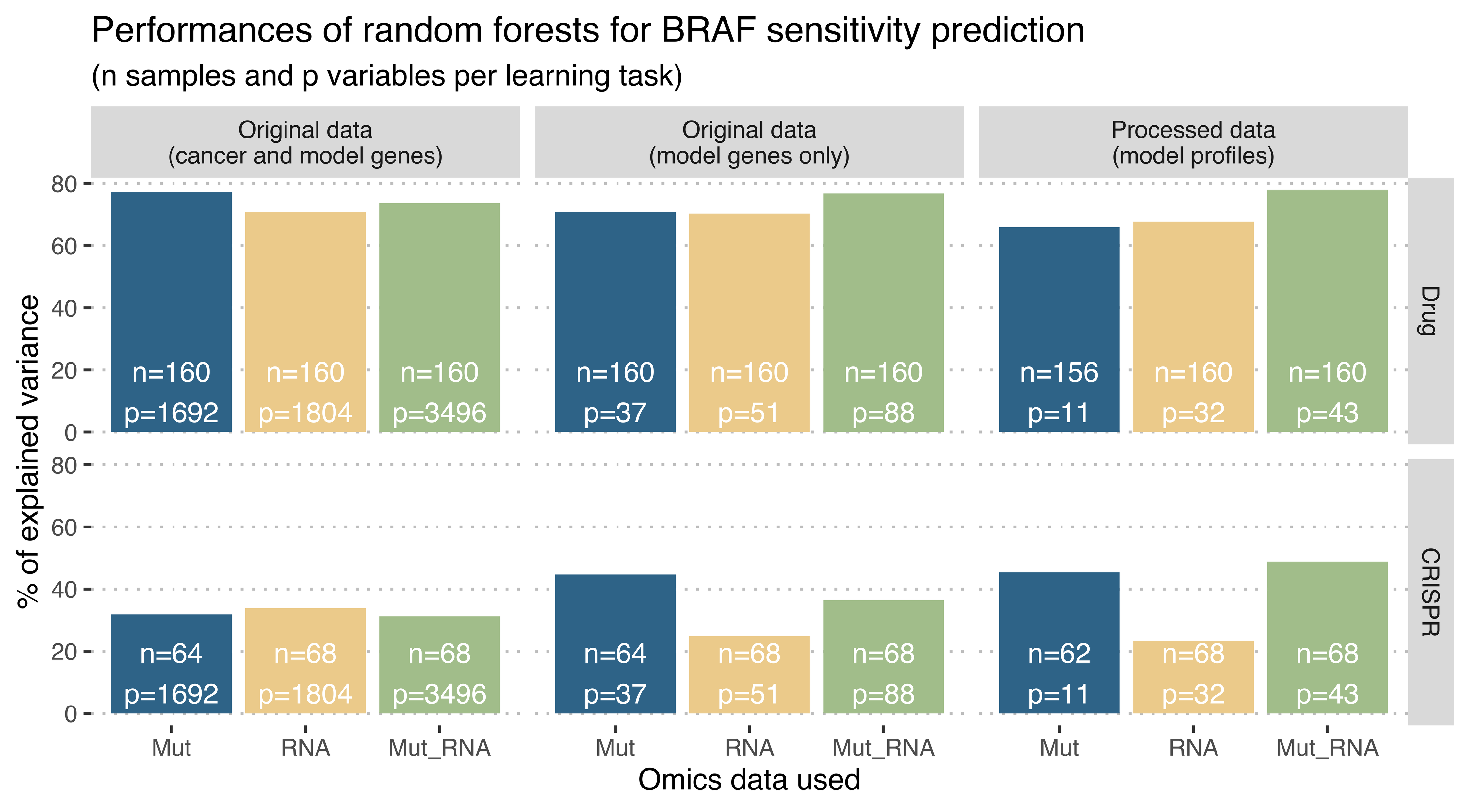

Supplement: S2 Fig — Random forests algorithms are trained with different omics types (mutations, RNA or both) and data processing (original data or processed data) to predict sensitivity to BRAF inhibition, through drug or CRISPR screening. Performances are expressed as percentage of explained variance by the fitted random forests. (TIF) [file pcbi.1007900.s002.tif]

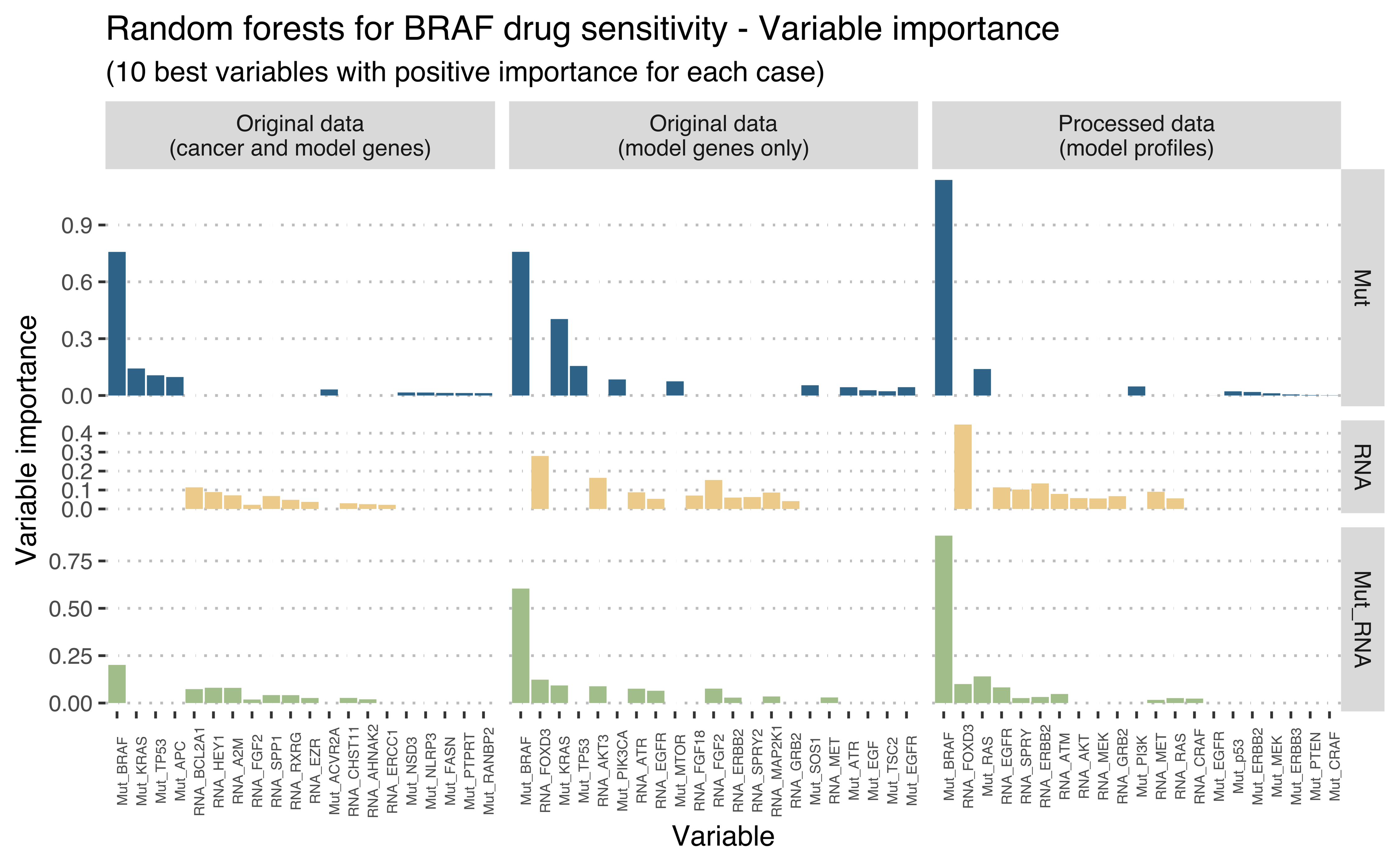

Supplement: S3 Fig — Variable importance for inhibition of BRAF by drugs (first row in S2 Fig), when random forests algorithms are trained with different omics types (mutations, RNA or both) and data processing (original data or processed data). Higher values of variable importance correspond to higher decrease in prediction performance when the variable is disturbed by permutation and therefore to variables with a positive contribution to predictive performance. (TIF) [file pcbi.1007900.s003.tif]
